# Accounting for trends in health poverty: a decomposition analysis for Britain, 1991–2008

**DOI:** 10.1007/s10198-014-0561-0

**Published:** 2014-01-22

**Authors:** Michal Brzezinski

**Affiliations:** Faculty of Economic Sciences, University of Warsaw, Dluga 44/50, 00-241 Warsaw, Poland

**Keywords:** Health poverty, Ordinal FGT measures, Self-reported health, Statistical inference, British Household Panel Survey, I32, I1, D63

## Abstract

We use data from the British Household Panel Survey to analyse changes in poverty of self-reported health from 1991 to 2008. We use the indices recently introduced by Bennett and Hatzimasoura (Poverty measurement with ordinal data. Institute for International Economic Policy, IIEP-WP-2011-14, 2011), which can be interpreted as ordinal counterparts of the classical Foster et al. (Econometrica 52(3):761–766, 1984) poverty measures. We decompose changes in self-reported health poverty over time into within-group health poverty changes and population shifts between groups. We also provide statistical inference for the Bennett and Hatzimasoura’s (Poverty measurement with ordinal data. Institute for International Economic Policy, IIEP-WP-2011-14, 2011) indices. Results suggest that when “fair” self-reported health status is chosen as a health poverty threshold all of the used indices indicate the growth of health poverty in Britain. However, when the health poverty threshold is lower (“poor” self-reported health status) the increase in health poverty incidence was compensated by decreasing average health poverty depth and improving health inequality among those who are poor with respect to health. The subgroup decompositions suggest that the most important factors accounting for the changes in total health poverty in Britain include a rise of both health poverty and population shares of persons cohabiting and couples with no children as well as an increase of the population of retired persons.

## Introduction

In recent years there has been a growing interest in analysing the distribution of self-rated health statuses in a population and its changes over time. The problem that has received most attention is the appropriate measurement of health inequality that accounts for the ordinal nature of self-reported data (see, e.g., [1, 2, 5, 17]). A related, but different distributional problem of health *poverty* has been less studied.^1^ As noticed by Allison and Foster [2], the most popular poverty measure using self-rated data is poverty headcount rate defined as the proportion of a population whose health status is below a chosen threshold.^2^ In case of studies using data based on a five-point scale of self-assessed health with categories of “poor”, “fair”, “good”, “very good” and “excellent”, the health poverty headcount rate has been usually defined as the share of population with poor or fair health. However, such a simple measure takes into account only poverty incidence, but it is insensitive to poverty depth and distribution among the poor (poverty severity) as it weights respondents with poor and fair health equally. Poverty measurement literature delivers several families of poverty indices which are sensitive to the poverty incidence, depth and severity—most notably the Foster, Greer, Thorbecke (FGT) family, introduced in Foster et al. [7]. The FGT indices are, however, designed for cardinally measurable and interpersonally comparable variables like income and they are not meaningful when applied to ordinal data like self-rated health statuses [8].^3^ The main reason for this is that they are not invariant to order-preserving transformations applied to the numerical values representing self-reported health statuses and the poverty threshold. To overcome this difficulty, Bennett and Hatzimasoura [3] recently proposed ordinal counterparts of the FGT poverty measures, which are invariant to order-preserving transformations and possess many attractive features of the original FGT measures. From the policy perspective, the most attractive feature of the FGT indices, both the original ones and their ordinal counterparts, is their subgroup decomposability. This property means that for any division of the population into nonoverlapping subgroups, total poverty measured by an FGT index can be expressed as a sum of the subgroup poverty indices weighted with population shares of subgroups.^4^ The ordinal FGT indices of Bennett and Hatzimasoura [3] can be therefore used to identify the subgroups which are more affected by health poverty and to design policies that may be most effective in reducing overall health poverty.

The purpose of this paper is to analyse trends in self-reported health poverty in Britain using ordinal FGT measures of Bennett and Hatzimasoura [3] and data from the British Household Panel Survey (BHPS) for the period between 1991 and 2008. We also provide statistical inference for these ordinal FGT indices to verify if the observed changes in health poverty are due to sampling variability or if they correspond to the true changes in the population. Finally, we borrow from the literature on decomposing poverty indices using the Shapley value concept [12] to provide decompositions of changes in total self-rated health poverty in Britain between 1991 and 2008 into changes in subgroups’ population shares and changes in health poverty levels within subgroups.

## Measures of self-rated health poverty

Bennett and Hatzimasoura’s [3] ordinal FGT family of poverty indices may be defined in the context of self-rated health data as follows. Let self-rated health of a population consisting of *n* persons be represented by a vector of *S* ordered categories *Y* = (*y*_1_, *y*_2_,…, *y*_*S*_), with *y*_*i*_ > *y*_*j*_ if and only if health status *i* is preferred to health status *j*. In practice *y*_1_ may represent, for example, poor self-rated health status, while *y*_*S*_ may represent excellent self-rated health status. If category *k* is chosen as a poverty threshold, then Bennett and Hatzimasoura [3] propose the following class of ordinal poverty measures:

1 $$ \pi_{\alpha } (Y;k) = \sum\limits_{j = 1}^{k} {p_{j} \left( {\frac{k - j + 1}{k}} \right)^{\alpha } ,} $$

where *p*_*j*_ is the share of population with self-rated health *y*_*j*_ and *α* ≥ 0 is a parameter. Notice that *p*_*j*_ can be interpreted as a probability of having self-rated health *y*_*j*_ and hence Eq. (1) can be viewed as a weighted sum of the probabilities of having self-rated health below the chosen health poverty threshold with weights determined by *k* (the number of self-rated health categories below or equal to the poverty threshold) and the parameter *α*. If *α* = 0, then Eq. (1) reduces to the standard poverty headcount rate, while if *α* > 0, then Eq. (1) gives more weight to the categories with lower self-rated health. For example, when *k* = 2 and *α* = 1, the weights for *p*_1_ and *p*_2_ are, respectively, 1 and 1/2. Higher values of parameter *α* lead to lower weights attached to *p*_2_,…, *p*_*k*_. Using an alternative representation of Eq. (1) in terms of normalized health ranks, Bennett and Hatzimasoura [3] show that the ordinal FGT measures are sensitive both to depth (when *α* > 0) and depth and distribution (when *α* > 1) of health poverty.

### Statistical inference

The family of ordinal FGT poverty indices (1) is a linear function of *k* population parameters, *p* = (*p*_1_,…, *p*_*k*_)^T^. In particular, it takes the form

2 $$ \pi_{\alpha } (Y;k) = cp, $$

with $c = \left[ {1,\left( {\frac{k - 1}{k}} \right), \ldots ,\left( \frac{1}{k} \right)^{\alpha } } \right]$. For a random sample of *n* individuals, the maximum likelihood estimator of a population share *p*_*i*_ is simply the sample proportion, $\hat{p}_{i} = a_{i} /n$, where *a*_*i*_ is the number of persons with self-rated health status *y*_*i*_ in the sample. The maximum likelihood estimator of $\pi_{\alpha } (Y;k)$ is therefore given by

3 $$ \hat{\pi }_{\alpha } (Y;k) = c\hat{p}, $$

where $\hat{p}$ is a column vector of sample estimates of *p*_*i*_. From the central limit theorem, $\hat{\pi }_{\alpha } (Y;k)$ is (asymptotically) normally distributed with a covariance matrix, which can be obtained using the delta method. The covariance matrix of *p* is given by

4 $$ \sum { = \frac{1}{n}} \left[ {\begin{array}{*{20}c} {p_{1} (1 - p_{1} )} & { - p_{1} p_{2} } & \ldots & { - p_{1} p_{k} } \\ { - p_{2} p_{1} } & {p_{2} (1 - p_{2} )} & \ldots & { - p_{2} p_{k} } \\ \vdots & \vdots & \vdots & \vdots \\ { - p_{k} p_{1} } & { - p_{k} p_{2} } & \ldots & {p_{k} (1 - p_{k} )} \\ \end{array} } \right]. $$

Therefore, the variance of Eq. (2) is given by

5 $$ {\text{Var}}\left( {\pi_{\alpha } (Y;k)} \right) = c\sum {c^{\text{T}} } . $$

The sample estimate of Eq. (5), $\widehat{\text{Var}}(\hat{\pi }_{\alpha } (Y;k)),$ can be obtained by replacing in Eq. (4) each *p*_*i*_ by its sample estimate $\hat{p}_{i} .$

The variance estimator of $\hat{\pi }_{\alpha } (Y;k)$ can be used to construct confidence intervals for estimated self-rated health poverty indices and to test hypotheses about the estimated indices. In particular, in order to test the hypothesis that two distributions of self-rated health, *X* and *Y*, have the same value of a given ordinal FGT index, we may use the following statistic:

6 $$ \tau = \frac{{\hat{\pi }_{\alpha } (X;k) - \hat{\pi }_{\alpha } (Y;k)}}{{\sqrt {\widehat{\text{Var}}\left( {\hat{\pi }_{\alpha } (X;k)} \right) + \widehat{\text{Var}}\left( {\hat{\pi }_{\alpha } (Y;k)} \right) - 2\widehat{\text{Cov}}\left( {\hat{\pi }_{\alpha } (X;k),\hat{\pi }_{\alpha } (Y;k)} \right)} }}. $$

If the samples *X* and *Y* are independent, the covariance term in the denominator of Eq. (6) is zero. However, the samples taken from two different waves of the BHPS are dependent as the BHPS is a longitudinal survey, which interviews annually the same individuals belonging to a representative sample chosen in 1991. The dependence of two BHPS samples taken from two different survey waves is only partial owing to sample attrition and inclusion of new entrants after wave 1 (see [13]). An appropriate method of accounting for partial sample dependency was proposed by Zheng [16] in the context of the inference for continuous additively separable poverty measures (including the continuous FGT indices).^5^ In this paper, we use Zheng’s [16] approach to calculate the covariance term in Eq. (6).

### Subgroup decomposition of changes in self-reported health poverty over time

In order to identify how various subgroups contribute to changes in self-reported health poverty over time, we can use “dynamic” decompositions of poverty changes proposed in the distributional literature concerned with continuous outcome variables. For subgroup decomposable ordinal FGT measures defined in Eq. (1), changes in total poverty over time from *t*_1_ to *t*_2_ can be written as follows:

7 $$ \Delta \pi_{\alpha } = \pi_{\alpha } (Y_{{t_{2} }} ;k) - \pi_{\alpha } (Y_{{t_{1} }} ;k) = \sum\limits_{i = 1}^{h} {\left[ {v^{i} (t_{2} )\pi_{\alpha }^{i} (Y_{{t_{2} }} ;k) - v^{i} (t_{1} )\pi_{\alpha }^{i} (Y_{{t_{1} }} ;k)} \right],} $$

where *v* ^*i*^ and $\pi_{\alpha }^{i}$ are, respectively, population share and poverty level of subgroup *i* ∈ (1,…, *h*). Accounting for the change in total poverty over time, Δ*π*_*α*_ can be expressed in terms of changes in poverty within subgroups, $\Delta \pi_{\alpha }^{i} = \pi_{\alpha }^{i} (Y_{{t_{2} }} ;k) - \pi_{\alpha }^{i} (Y_{{t_{1} }} ;k),$ *i* ∈ (1,…, *h*), and changes in population shares of subgroups, $\Delta v^{i} = v^{i} (t_{2} ) - v^{i} (t_{1} ),$ *i* ∈ (1,…, *h*). Shorrocks [12] has shown that an exact decomposition of this kind can be performed using the Shapley value concept taken from the cooperative game theory.^6^ According to the Shapley value based decomposition, Eq. (7) becomes

8 $$ \Delta \pi_{\alpha } = \sum\limits_{i = 1}^{h} {\left( {W^{i} + p^{i} } \right) = \sum\limits_{i = 1}^{h} {\left[ {\frac{{v^{i} (t_{1} ) + v^{i} (t_{2} )}}{2}\Delta \pi_{\alpha }^{i} + \frac{{\pi_{\alpha }^{i} \left( {Y_{{t_{1} }} ;k} \right) + \pi_{\alpha }^{i} \left( {Y_{{t_{2} }} ;k} \right)}}{2}\Delta v^{i} } \right]} .} $$

Within-subgroup effects, *W* ^*i*^, measure the contribution of poverty changes within subgroups to changes in total poverty weighted by the subgroups’ population shares averaged over time. Between-subgroup population shift effects, *P* ^*i*^, are defined as contributions of changes in subgroups’ population shares to changes in total poverty weighted by the subgroup levels of poverty averaged over time. A poverty change decomposition similar to that given by Eq. (8), but with weights coming from the initial period (*t*_1_), was initially proposed by Ravallion and Huppi [11]. However, their decomposition was inexact as it contained an interaction term between $\Delta \pi_{\alpha }^{i} \,{\text{and}}\,\Delta v^{i}$. Shapley value based decomposition in Eq. (8) does not suffer from this drawback.

## Data

We use data from waves 1–18 of the BHPS. The BHPS was designed as a nationally representative annual survey of the adult (aged 16+) population of Great Britain [13]. It re-interviews annually the same individuals belonging to the initial sample of more than 5,000 households as well as their adult co-residents. The BHPS collects rich information about respondents’ household structure, health, incomes, labour market status, housing conditions, education and socio-economic values. In this paper, we are mainly interested in cross-sectional analysis of trends in self-reported health in Britain. For this reason, we use information on all respondents giving the full interview in a given year weighted with cross-sectional weights available in the BHPS that adjust for inclusion of new entrants and for within household nonresponse. We also use information about clustering and stratification of the BHPS sample (see [13]) in estimating covariance matrix Σ in Eq. (3). The total number of observations ranges from 9,790 in 1991 to 7,125 in 2008.

The self-rated health status is measured in the BHPS using an answer to the question: “Please think back over the last 12 months about how your health has been. Compared to people of your own age, would you say your health has on the whole been excellent, good, fair, poor or very poor?”^7^ Table 1 presents the distribution of self-rated health for 1991 and 2008. For the purposes of decomposing health poverty we use also information on individual marital status, household type and labour market status. The distributions of these variables in 1991 and 2008 are given in Table 3.

**Table 1:** Distribution of self-rated health status for the BHPS data, percent of samples

Self-assessed health status	1991	2008
Excellent	28.1	20.3
Good	45	47.7
Fair	18.6	22.5
Poor	6.2	7.7
Very poor	2.1	1.7

Estimates are weighted with cross-sectional respondent weights

## Poverty of self-reported health in Britain, 1991–2008

### Trends in self-rated health poverty

**Fig. 1:**
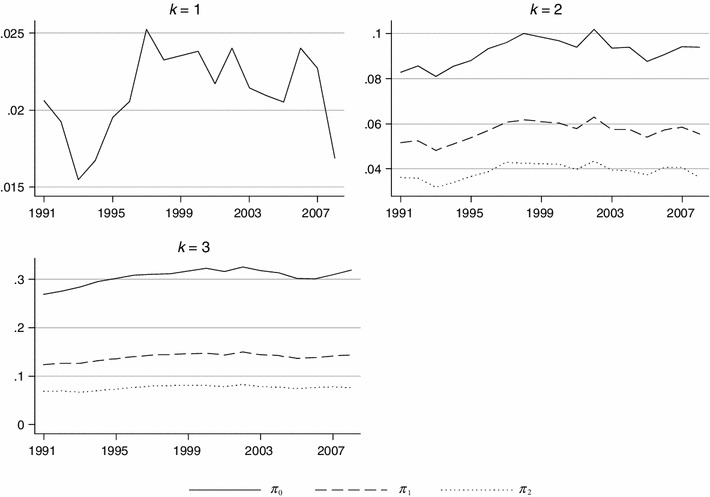
Trends in ordinal FGT poverty indices for the BHPS data with different health poverty thresholds (*k* = 1, 2, 3)

Figure 1 shows trends in poverty of self-rated health using ordinal FGT indices of Bennett and Hatzimasoura [3] with different values of *α* and different poverty thresholds *k*. The lowest possible poverty threshold *k* = 1 is certainly unreasonable as people reporting higher self-rated health status still consider it to be “poor”.

For more reasonable poverty thresholds, we observe that health poverty as measured by poverty headcount rate ($\pi_{0}$) increased between 1991 and 2008 by 13.7 % and by 18.9 % for *k* = 2 (“poor” self-rated health status) and *k* = 3 (“fair” self-rated health status), respectively. The growth of health poverty was smaller in the case of $\pi_{1}$—7.2 % (*k* = 2) and 15.6 % (*k* = 3). Finally, self-reported health poverty as measured by $\pi_{2}$ did not change when *k* = 2 and increased by 10.6 % when *k* = 3. Table 2 presents estimates of health poverty indices for *k* = 2 and 3 together with their standard errors and 95 % confidence intervals.^8^ It also gives results of significance tests on pairwise health poverty comparisons between 1991 and 2008.

**Table 2:** Ordinal FGT indices for self-assessed health status (*k* = 2, 3)

	*π*_0_	*π*_1_	*π*_2_
*k* = 2			
1991	0.0827 (0.0030) [0.0769, 0.0885]	0.0517 (0.0020) [0.0478, 0.0556]	0.0361 (0.0016) [0.0329, 0.0394]
2008	0.0940 (0.0043) [0.0855, 0.1025]	0.0554 (0.0027) [0.0501, 0.0608]	0.0362 (0.0021) [0.0320, 0.0403]
2008 versus 1991	0.0113 (0.0050) 0.026	0.0038 (0.0032) 0.245	0.0000 (0.0026) 0.989
*k* = 3			
1991	0.2686 (0.0053) [0.2582, 0.2790]	0.1240 (0.0026) [0.1188, 0.1292]	0.0689 (0.0019) [0.0651, 0.0726]
2008	0.3193 (0.0071) [0.3055, 0.3331]	0.1434 (0.0037) [0.1361, 0.1507]	0.0762 (0.0027) [0.0710, 0.0814]
2008 versus 1991	0.0507 (0.0085) 0.000	0.0194 (0.0044) 0.000	0.0073 (0.0031) 0.021

Standard errors appear in parentheses, 95 % normal-based confidence intervals are given in square brackets. Rows for pairwise comparisons give a difference in poverty indices as well as its standard error and associated *p* value corrected for sample dependency

The results suggest that for *k* = 2 a change in self-rated health poverty headcount is significant at the conventional 5 % significance level. However, if measures sensitive to depth ($\pi_{1}$) and depth and distribution of poverty ($\pi_{2}$) are applied, the results for *k* = 2 become statistically insignificant. This means that the observed increase in health poverty incidence as measured by $\pi_{0}$ was accompanied by both the decrease in average health poverty depth and the decrease in inequality of health poverty. These additional insights would not be gained if health poverty was measured using poverty headcount index only.

When an even higher poverty threshold is used (*k* = 3), health poverty increases displayed by all poverty indices used are statistically significant.

### Decomposition of health poverty changes

Table 3 presents results of subgroup decompositions of changes in self-rated health poverty in Britain between 1991 and 2008 when health poverty is measured by $\pi_{2}$ with *k* = 3.^9^ The total change in health poverty, denoted by *δ*, is 0.0073 or 10.6 % in relative terms. We perform decompositions for subgroups defined by marital status, household type and labour market status.^10^

**Table 3:** Subgroup decompositions of changes in *π*_2_ for self-reported health (*k* = 3)

Group	1991		2008		1991–2008	
	*v*	*π*_2_	*v*	*π*_2_	*W*	*P*
Marital status						
Married	58.4	0.064	52.8	0.070	45.7	−51.8
Cohabiting	6.3	0.049	10.4	0.069	22.6	33.1
Widowed	9.1	0.128	8.2	0.147	22.7	−15.9
Divorced/separated	5.7	0.120	7.3	0.126	5.2	26.3
Single never married	20.5	0.049	21.3	0.051	6.3	5.7
Total population	100	0.069	100	0.076	102.5	−2.5
Household type						
Single non-elderly (age <65)	5.9	0.101	7.8	0.092	−8.6	25.9
Single elderly (age 65+)	7.6	0.123	8.6	0.137	15.5	18.3
Couple with no children	39.8	0.073	42.5	0.079	35.1	27.4
Couple with children	24.8	0.046	20.6	0.050	14.1	−27.8
Lone parent	2.0	0.074	1.9	0.130	14.7	−0.6
Other households	19.9	0.059	18.6	0.058	−3.1	−10.6
Total population	100	0.069	100	0.076	67.7	32.3
Labour market status						
Full-time employee	38.7	0.038	38.0	0.042	20.8	−3.6
Part-time employee	9.9	0.040	10.8	0.044	5.8	5.2
Self-employed	7.7	0.028	7.2	0.041	13.7	−2.6
Unemployed	5.5	0.058	3.0	0.078	11.2	−23.5
Retired	19.5	0.111	25.9	0.113	7.7	97.5
Inactive	18.7	0.124	15.1	0.138	30.8	−63.0
Total population	100	0.069	100	0.076	90.0	10.0

*W* and *P* represent, respectively, the within-subgroup and the between-subgroup contributions to the total health poverty changes. They are expressed as percentages of changes in total health poverty

The decomposition based on marital status suggests that between-subgroup population shifts had overall an offsetting effect on changes in total poverty. The largest overall poverty-increasing effect among subgroups is due to increasing health poverty and population share of persons cohabiting. Turning to decompositions using subgroups defined by household type, we note that the within-subgroup population shifts accounted for as much as about 32 % of *δ*. Increases in the populations of single non-elderly persons and couples with no children each contributed to more than 25 % of *δ*. Health poverty increase among couples with no children accounted for about 35 % of the overall health poverty change, while a fall of health poverty among single non-elderly persons had a rather small poverty-decreasing effect. Finally, in case of decomposition for subgroups defined by labour market status 90 % of *δ* can be accounted for by within-subgroups poverty effects. However, detailed analysis of population shift effects reveals interesting facts. The population of retired persons in the BHPS increased between 1991 and 2008 from 19.5 % to 25.9 %, which accounts for as much as 97.5 % of the total health poverty increase. This large effect is, however, almost offset by significant decreases in the populations of inactive and unemployed persons. The biggest contributions to *δ* among the within-subgroup poverty effects can be assigned to deterioration in health among inactive persons (30.8 %) and full-time employees (20.8 %).

## Conclusions

This paper used data from the BHPS to provide an analysis of trends in self-rated health poverty in Britain over 1991–2008. We used ordinal FGT poverty indices proposed recently by Bennett and Hatzimasoura [3], which are appropriate for the ordinal nature of self-rated health data. We have also extended the approach of Bennett and Hatzimasoura [3] by providing statistical inference for their ordinal FGT indices. Moreover, we have used the subgroup decompositions of health poverty changes borrowed from the literature on measuring income poverty.

Our results suggest that empirically there are additional insights from analysing health poverty with Bennett and Hatzimasoura’s [3] family of ordinal FGT indices, rather than using health poverty headcount rate only. The BHPS data show that when “fair” self-reported health status is chosen as a health poverty threshold all of the used ordinal FGT indices indicate the growth of health poverty in Britain. However, when health poverty threshold is lower (“poor” self-reported health status) only poverty headcount rate increases in a statistically significant way. For this threshold, the observed increase in health poverty incidence was accompanied by decreasing average health poverty depth and improving health inequality among those who are poor with respect to health.

More generally, we may expect that the ordinal FGT poverty indices of Bennett and Hatzimasoura [3] may be also useful in analysing data with more levels of self-reported health statuses. For example, it would be interesting to check if trends in poverty of *satisfaction with health*, which is measured in practice even on a 11-point ordinal scale (see, e.g., [9]), are robust to the choice of a poverty threshold.

## Footnotes

1 In this paper, we use self-reported health as an indicator of overall health. Therefore, by “health poverty” we mean “self-reported health poverty”.

2 Another popular approach to analyse health poverty with self-reported data is based on translating ordinal information into cardinal information (see, e.g., [10] using methods established in health economics literature [14].

3 The only exception is poverty headcount rate, which is a member of the FGT class with poverty aversion parameter set to 0. However, as stated before, the poverty headcount rate is not sensitive to poverty depth and severity.

4 See Chakravarty [4], for a recent overview of various poverty indices and their properties.

5 See also Zheng and Cushing [15] for the same procedure applied to inference on inequality with dependent samples.

6 For a textbook treatment of Shapley value based decompositions of poverty and inequality, see Duclos and Araar [6].

7 We do not include wave 9 of the BHPS in our analysis as there was a change in wording of the self-rated health question in this wave.

8 The health poverty change between 1991 and 2008 for *k* = 1 is 0.0037, which is not statistically significant with a *p* value of 0.116.

9 Results for $\pi_{0}$ and $\pi_{1}$ with *k* = 3 are in general qualitatively similar to those for $\pi_{2}$ (*k* = 3).

10 Decompositions for subgroups defined by the number of children, education and income group are available upon request.
